# Supplementary material for: Impacts of yeast Tma20/MCTS1, Tma22/DENR and Tma64/eIF2D on translation reinitiation and ribosome recycling
Source: bioRxiv. 2024 Mar 7:2024.03.06.583729. Preprint. [Version 1] doi: 10.1101/2024.03.06.583729 (PMC11188067; doi:10.1101/2024.03.06.583729)
Supplement: Supplement 1 [file NIHPP2024.03.06.583729v1-supplement-1.pdf]

bioRxiv preprint doi: https://doi.org/10.1101/2024.03.06.583729; this version posted March 7, 2024. The copyright holder for this preprint (which was not certified by peer review) is the author/funder. This article is a US Government work. It is not subject to copyright under 17 USC 105 and is also made available for use under a CC0 license.

SUPPLEMENTAL INFORMATION

Impacts of yeast Tma20/MCTS1, Tma22/DENR and Tma64/eIF2D on translation reinitiation and ribosome recycling.

Kristína Jendruchová[1,2#], Swati Gaikwad[3#], Kristýna Poncová[1], Stanislava Gunišová[1], Leoš Shivaya Valášek[1*], and Alan G. Hinnebusch[3*]

[1]Laboratory of Regulation of Gene Expression, Institute of Microbiology of the Czech Academy of Sciences, Videnska 1083, 142 20, Prague, the Czech Republic

[2]Faculty of Science, Charles University, Albertov 6, 128 00 Prague, Czech Republic

[3]Divsion of Molecular and Cellular Biology, Eunice Kennedy Shriver National Institute of Child Health and Human Development, National Institutes of Health, Bethesda, MD 20892, USA

*Corresponding authors: valasekl@biomed.cas.cz, alanh@mail.nih.gov
[#]These authors contributed equally to this work.

Running title: Tma proteins in reinitiation and ribosome recycling

Keywords: Tma, MCTS1, DENR, eIF2D, reinitiation, ribosome recycling

bioRxiv preprint doi: https://doi.org/10.1101/2024.03.06.583729; this version posted March 7, 2024. The copyright holder for this preprint (which was not certified by peer review) is the author/funder. This article is a US Government work. It is not subject to copyright under 17 USC 105 and is also made available for use under a CC0 license.

SUPPLEMENTARY TABLES

Table S1. Yeast strains used in this study.

This table lists all yeast strains employed in this study along with their respective parental strains used to generate them. The details of strain constructions are described in MATERIALS AND METHODS.

**Name:**	**Genotype:**	**Parental strain:**	**Source:**
YSG142 (WT BY4741)	*MATa his3Δ1 leu2Δ0 met15Δ0 ura3Δ0*	-	Euroscarf
YSG178 (*Δtma64*)	*MATa his3Δ1 leu2Δ0 met15Δ0 ura3Δ0 ydr117CΔ:: kanMX4*	YSG142	This study
YSG181 (*Δtma20*)	*MATa his3Δ1 leu2Δ0 met15Δ0 ura3Δ0 yer007C-AΔ::kanMX4*	YSG142	Euroscarf
YSG184 (*Δtma22*)	*MATa his3Δ1 leu2Δ0 met15Δ0 ura3Δ0 yjr014WΔ::kanMX4*	YSG142	Euroscarf
YSG196 (*Δtma20 Δtma64*)	*MATa his3Δ1 leu2Δ0 met15Δ0 ura3Δ0 yer007C-AΔ::kanMX4 ydr117CΔ::natNT2*	YSG181	This study
YKJ3 (*Δtma20 Δtma22 Δtma64*)	*MATa his3Δ1 leu2Δ0 met15Δ0 ura3Δ0 yer007C-AΔ::kanMX4 ydr117CΔ::natNT2 yjr014WΔ::hphNT1*	YSG196	This study
YKJ6 (*Δtma20 Δtma22*)	*MATa his3Δ1 leu2Δ0 met15Δ0 ura3Δ0 yer007C-AΔ::kanMX4 yjr014WΔ::natNT2*	YSG181	This study
BY4741	*MATa his3Δ1 leu2Δ0 met15Δ0 ura3Δ0*	-	
H4520	*MATa his3Δ1 leu2Δ0 met15Δ0 ura3Δ0 ydr117CΔ::hygMX4; yer007C-AΔ::kanMX4*	BY4741	[1]

bioRxiv preprint doi: https://doi.org/10.1101/2024.03.06.583729; this version posted March 7, 2024. The copyright holder for this preprint (which was not certified by peer review) is the author/funder. This article is a US Government work. It is not subject to copyright under 17 USC 105 and is also made available for use under a CC0 license.

Table S2. Plasmids used in this study.
This table lists all plasmids used in this study, with construction details described in MATERIALS AND METHODS.

Plasmid:	Description:	Reference:
p227 (uORF-less)	low copy *URA3 GCN4-lacZ* plasmid containing the uORF-less *GCN4* leader created by point mutations in uORF1 (*Hind*III), uORF2 (*Eco*RI), uORF3 (*Kpn*I) and uORF4 (*Bgl*II)	[2]
p226 (uORF4-only)	low copy *URA3 GCN4-lacZ* plasmid containing *GCN4* leader with uORF4 only at its original position	[2]
pKJ34	p226 with uORF4 penultimate codon mutated to TTG	This study
pKJ35	p226 with uORF4 penultimate codon mutated to ATT	This study
pKJ36	p226 with uORF4 penultimate codon mutated to TGG	This study
pKJ57	p226 with uORF4 penultimate codon mutated to CAA	This study
pKJ58	p226 with uORF4 penultimate codon mutated to GCT	This study
pKJ59	p226 with uORF4 penultimate codon mutated to TAT	This study
pKJ61	p226 with uORF4 penultimate codon mutated to AAT	This study
pKJ62	p226 with uORF4 penultimate codon mutated to CCA	This study
pKJ23	p226 with uORF4 penultimate codon mutated to ATG	This study
pKJ20	p226 with uORF4 penultimate codon mutated to TAC	This study
pKJ21	p226 with uORF4 penultimate codon mutated to GTG	This study
pKP76	low copy *URA3 GCN4-lacZ* plasmid containing uORF3 only at its original position mutated into a start-stop element	This study
pKP77	low copy *URA3 GCN4-lacZ* plasmid containing uORF4 only at its original position mutated into a start-stop element	This study
pKP78	low copy *URA3 GCN4-lacZ* plasmid containing uORF1 only at its original position mutated into a start-stop element	This study

bioRxiv preprint doi: https://doi.org/10.1101/2024.03.06.583729; this version posted March 7, 2024. The copyright holder for this preprint (which was not certified by peer review) is the author/funder. This article is a US Government work. It is not subject to copyright under 17 USC 105 and is also made available for use under a CC0 license.

pSG194	low copy *URA3 GCN4-lacZ* plasmid containing an an uORF1 variant only with coding sequence replaced by the corresponding sequence of uORF2	[3]
pKJ17	p226 with uORF4 penultimate codon mutated to CTG	This study
pFA6a-KanMX4	pFA6 backbone containing *KanMX4* resistance marker-based deletion cassette	[4]
pZC3	pFA6 backbone containing *hphNT1* resistance marker-based deletion cassette	[5]
pZC4	pFA6 backbone containing *natNT2* resistance marker-based deletion cassette	[5]
pSG61	low copy *URA3 GCN4-lacZ* plasmid containing the *GCN4* leader with uORF1 only at its original position	This study
pSG62	pSG61 with uORF1 penultimate codon mutated to ATT	This study
pSG63	pSG61 with uORF1 penultimate codon mutated to TTG	This study
pSG64	pSG61 with uORF1 penultimate codon mutated to AAA	This study
pSG65	pSG61 with uORF1 penultimate codon mutated to AAT	This study
pSG66	pSG61 with uORF1 penultimate codon mutated to AAG	This study
pSG67	pSG61 with uORF1 penultimate codon mutated to TAT	This study
pSG68	pSG61 with uORF1 penultimate codon mutated to ATG	This study
pSG69	pSG61 with uORF1 penultimate codon mutated to GCG	This study
pSG70	pSG61 with uORF1 penultimate codon mutated to CTG	This study
pSG71	pSG61 with uORF1 penultimate codon mutated to TGG	This study
pSG72	pSG61 with uORF1 penultimate codon mutated to TAC	This study
pSG73	pSG61 with uORF1 penultimate codon mutated to CAA	This study
pSG74	pSG61 with uORF1 penultimate codon mutated to GAC	This study
pSG75	pSG61 with uORF1 penultimate codon mutated to GAG	This study

bioRxiv preprint doi: https://doi.org/10.1101/2024.03.06.583729; this version posted March 7, 2024. The copyright holder for this preprint (which was not certified by peer review) is the author/funder. This article is a US Government work. It is not subject to copyright under 17 USC 105 and is also made available for use under a CC0 license.

pSG76	pSG61 with uORF1 penultimate codon mutated to TGT	This study
pSG77	pSG61 with uORF1 penultimate codon mutated to GCT	This study
pSG78	pSG61 with uORF1 penultimate codon mutated to CAC	This study
pSG79	pSG61 with uORF1 penultimate codon mutated to TTT	This study
pSG80	pSG61 with uORF1 penultimate codon mutated to GAA	This study
pSG81	pSG61 with uORF1 penultimate codon mutated to CGC	This study
pSG82	pSG61 with uORF1 penultimate codon mutated to GTC	This study
pSG83	pSG61 with uORF1 penultimate codon mutated to GGG	This study
pSG84	pSG61 with uORF1 penultimate codon mutated to CCG	This study
pSG85	pSG61 with uORF1 penultimate codon mutated to CGG	This study
pSG86	pSG61 with uORF1 penultimate codon mutated to CCC	This study
pSG87	pSG61 with uORF1 penultimate codon mutated to TCG	This study
pSG88	pSG61 with uORF1 penultimate codon mutated to AGG	This study
pSG89	pSG61 with uORF1 penultimate codon mutated to CCA	This study
pSG61 (2) (uORF3-only)	low copy *URA3 GCN4-lacZ* plasmid containing the *GCN4* leader with uORF3 only at its original position	[6]
p209 (uORF1-only)	low copy *URA3 GCN4-lacZ* plasmid containing the *GCN4* leader with uORF1 only at its original position	[7]

bioRxiv preprint doi: https://doi.org/10.1101/2024.03.06.583729; this version posted March 7, 2024. The copyright holder for this preprint (which was not certified by peer review) is the author/funder. This article is a US Government work. It is not subject to copyright under 17 USC 105 and is also made available for use under a CC0 license.

Table S3. Primers used in this study.
This table lists all primers used in this study.

**Primer:**	**Sequence:**
SG295	GAGAGTTGACCAATTACCTGACAGT
SG294	CTTGTAGCAAAGATTGGAAAAAGAG
SG296	ACTTGCACCATGTACATCAATTCTA
SG325	CTACTGAATAACCGACTCAATAGATTAGTGTAGCGCAGGATTAGTA CAGCTCTATAGAACGCGGCCGCCAG
SG326	GCTTTGATGTCGGGCATTTTTACGCATTTAAACATTTATATGATATAA ATCACTATAGGGAGACCGGCAG
KJ1	CCCAAGGAAACAGTTCAAGAGCTAAACTAAAGAAAAGCATATTGCA TAAACTATAGAACGCGGCCGCCAG
KJ2	GTAAAAAGTCCTTTTCTCCCAGAACGGTGCTATTACATATTTATGGA TTGCCACTATAGGGAGACCGGCAG
PB238	GCA GCG AGG AGC CGT AAT
KJ27	AGGGCATCGGTCGACGGGGAATAAAG
KJ24 TGG	TTGACAGAAAGGTAACCGTTACCAAAACATC
KJ25 TTG	ATTTGACAGAAAGGTAACCGTTACAAAAACATC
KJ26 ATT	TTTGACAGAAAGGTAACCGTTAAATAAACATCTTG
KJ73 AAA	AGAAAGGTAACCGTTATTTAAACATC
KJ74 AAT	AGAAAGGTAACCGTTAATTAAAC
KJ75 AAG	CAGAAAGGTAACCGTTACTTAAA
KJ76 TAT	ACAGAAAGGTAACCGTTAATAAAACATC
KJ77 CCA	AGAAAGGTAACCGTTATGGAAACA
KJ78 CAA	CAGAAAGGTAACCGTTATTGAAAC A
KJ79 GAC	AGAAAGGTAACCGTTAGTCAAACAT
KJ80 GCT	AGAAAGGTAACCGTTAAGCAAACAT

bioRxiv preprint doi: https://doi.org/10.1101/2024.03.06.583729; this version posted March 7, 2024. The copyright holder for this preprint (which was not certified by peer review) is the author/funder. This article is a US Government work. It is not subject to copyright under 17 USC 105 and is also made available for use under a CC0 license.

Table S4. Synthetized DNA inserts used in this study.
This table lists the sequences of DNA fragments used for cloning that were synthesized through GeneArt Gene Synthesis (Thermo Fisher Scientific) or by LifeSct LLC. The table is formatted as follows: Insert name (construct it was used for); Construct sequence.

Insert (construct):	Sequence:
MFL (pKJ17)	GAGCGCGACGTAATACGACTCACTATAGGGCGAAT TGGCGGAAGGCCGTCAAGGCCGCATCAAGGGCATC GGTCGACGGGGAATAAAGTGCATGAGCATACATCTT GAAAAAAAAAGATGAAAAATTTCCGACTTTAAATACG GAAGATAAATACTCCAACCTTTTTTTCCAATTCCGAA ATTTTAGTCTTCTTTAAAGAAGTTTCGGCTCGCTGTC TTACCTTTTAAAATCTTCTACTTCTTGACAGTACTTAT CTTCTTATATAATAGATATACAAAACAAAACAAAACA AAAACTCACAACACAGGTTACTCTCCCCCCTAAATT CAAATTTTTTTTGCCCATCAGTTTCACTAGCGAATTA TACAACTCACCAGCCACACAGCTCACTCATCTACTT CGCAATCAAAACAAAATATTTTATTTTAGTTCAGTTTA TTAAGTTATTATCAGTATCGTATTAAAAAATTAAAGAT CATTGAAAAAAGCTTGCTAAACCGATTATATTTTGTT TTTAAAGTAGATTATTATTAGAAAATTATTAAGAGAAT TCTGTGTTAAATTTATTGAAAGAGAAAATTTATTTTCC CTTATTAATTAAAGTCCTTTACTTTTTTTGAAAACTGT CAGTTTTTTGAAGAGTTATTTGTTTTGTTACCAATTG CTATCAGGTACCCGTAGAATTTTATTCAAGATGTTTC TGTAACGGTTACCTTTCTGTCAAACTGGGCCTCATG GGCCTTCCGCTCACTGCCCGCTTTCCAGTCGGGAA ACCTGTCGTGCCA
MFM (pKJ23)	GAGCGCGACGTAATACGACTCACTATAGGGCGAAT TGGCGGAAGGCCGTCAAGGCCGCATCAAGGGCATC GGTCGACGGGGAATAAAGTGCATGAGCATACATCTT GAAAAAAAAAGATGAAAAATTTCCGACTTTAAATACG GAAGATAAATACTCCAACCTTTTTTTCCAATTCCGAA ATTTTAGTCTTCTTTAAAGAAGTTTCGGCTCGCTGTC TTACCTTTTAAAATCTTCTACTTCTTGACAGTACTTAT CTTCTTATATAATAGATATACAAAACAAAACAAAACA AAAACTCACAACACAGGTTACTCTCCCCCCTAAATT CAAATTTTTTTTGCCCATCAGTTTCACTAGCGAATTA TACAACTCACCAGCCACACAGCTCACTCATCTACTT CGCAATCAAAACAAAATATTTTATTTTAGTTCAGTTTA TTAAGTTATTATCAGTATCGTATTAAAAAATTAAAGAT CATTGAAAAAAGCTTGCTAAACCGATTATATTTTGTT TTTAAAGTAGATTATTATTAGAAAATTATTAAGAGAAT

bioRxiv preprint doi: https://doi.org/10.1101/2024.03.06.583729; this version posted March 7, 2024. The copyright holder for this preprint (which was not certified by peer review) is the author/funder. This article is a US Government work. It is not subject to copyright under 17 USC 105 and is also made available for use under a CC0 license.

	TCTGTGTTAAATTTATTGAAAGAGAAAATTTATTTTCC<br>CTTATTAATTAAAGTCCTTTACTTTTTTTGAAAACTGT<br>CAGTTTTTTGAAGAGTTATTTGTTTTGTTACCAATTG<br>CTATCAGGTACCCGTAGAATTTTATTCAAGATGTTTA<br>TGTAACGGTTACCTTTCTGTCAAACTGGGCCTCATG<br>GGCCTTCCGCTCACTGCCCGCTTTCCAGTCGGGAA<br>ACCTGTCGTGCCA
MFY (pKJ20)	GAGCGCGACGTAATACGACTCACTATAGGGCGAAT<br>TGGCGGAAGGCCGTCAAGGCCGCATCAAGGGCATC<br>GGTCGACGGGGAATAAAGTGCATGAGCATACATCTT<br>GAAAAAAAAAGATGAAAAATTTCCGACTTTAAATACG<br>GAAGATAAATACTCCAACCTTTTTTTCCAATTCCGAA<br>ATTTTAGTCTTCTTTAAAGAAGTTTCGGCTCGCTGTC<br>TTACCTTTTAAAATCTTCTACTTCTTGACAGTACTTAT<br>CTTCTTATATAATAGATATACAAAACAAAACAAAACA<br>AAAACTCACAACACAGGTTACTCTCCCCCCTAAATT<br>CAAATTTTTTTTGCCCATCAGTTTCACTAGCGAATTA<br>TACAACTCACCAGCCACACAGCTCACTCATCTACTT<br>CGCAATCAAAACAAAATATTTTATTTTAGTTCAGTTTA<br>TTAAGTTATTATCAGTATCGTATTAAAAAATTAAAGAT<br>CATTGAAAAAAGCTTGCTAAACCGATTATATTTTGTT<br>TTTAAAGTAGATTATTATTAGAAAATTATTAAGAGAAT<br>TCTGTGTTAAATTTATTGAAAGAGAAAATTTATTTTCC<br>CTTATTAATTAAAGTCCTTTACTTTTTTTGAAAACTGT<br>CAGTTTTTTGAAGAGTTATTTGTTTTGTTACCAATTG<br>CTATCAGGTACCCGTAGAATTTTATTCAAGATGTTTT<br>ACTAACGGTTACCTTTCTGTCAAACTGGGCCTCATG<br>GGCCTTCCGCTCACTGCCCGCTTTCCAGTCGGGAA<br>ACCTGTCGTGCCA
MFA (pKJ21)	GAGCGCGACGTAATACGACTCACTATAGGGCGAAT<br>TGGCGGAAGGCCGTCAAGGCCGCATCAAGGGCATC<br>GGTCGACGGGGAATAAAGTGCATGAGCATACATCTT<br>GAAAAAAAAAGATGAAAAATTTCCGACTTTAAATACG<br>GAAGATAAATACTCCAACCTTTTTTTCCAATTCCGAA<br>ATTTTAGTCTTCTTTAAAGAAGTTTCGGCTCGCTGTC<br>TTACCTTTTAAAATCTTCTACTTCTTGACAGTACTTAT<br>CTTCTTATATAATAGATATACAAAACAAAACAAAACA<br>AAAACTCACAACACAGGTTACTCTCCCCCCTAAATT<br>CAAATTTTTTTTGCCCATCAGTTTCACTAGCGAATTA<br>TACAACTCACCAGCCACACAGCTCACTCATCTACTT<br>CGCAATCAAAACAAAATATTTTATTTTAGTTCAGTTTA

bioRxiv preprint doi: https://doi.org/10.1101/2024.03.06.583729; this version posted March 7, 2024. The copyright holder for this preprint (which was not certified by peer review) is the author/funder. This article is a US Government work. It is not subject to copyright under 17 USC 105 and is also made available for use under a CC0 license.

	TTAAGTTATTATCAGTATCGTATTAAAAAATTAAAGAT CATTGAAAAAAGCTTGCTAAACCGATTATATTTTGTT TTTAAAGTAGATTATTATTAGAAAATTATTAAGAGAAT TCTGTGTTAAATTTATTGAAAGAGAAAATTTATTTTCC CTTATTAATTAAAGTCCTTTACTTTTTTTGAAAACTGT CAGTTTTTTGAAGAGTTATTTGTTTTGTTACCAATTG CTATCAGGTACCCGTAGAATTTTATTCAAGATGTTTG CGTAACGGTTACCTTTCTGTCAAACTGGGCCTCATG GGCCTTCCGCTCACTGCCCGCTTTCCAGTCGGGAA ACCTGTCGTGCCA
uORF1 st-st fragment (pKP78)	GCTCTCAAGGGCATCGGTCGACGGGGAATAAAGTG CATGAGCATACATCTTGAAAAAAAA AGATGAAAAATTTCCGACTTTAAATACGGAAGATAAA TACTCCAACCTTTTTTTCCAATT CCGAAATTTTAGTCTTCTTTAAAGAAGTTTCGGCTCG CTGTCTTACCTTTTAAAATCTTC TACTTCTTGACAGTACTTATCTTCTTATATAATAGATA TACAAAACAAAACAAAACAAAA ACTCACAACACAGGTTACTCTCCCCCCTAAATTCAA ATTTTTTTTGCCCATCAGTTTCAC TAGCGAATTATACAACTCACCAGCCACACAGCTCAC TCATCTACTTCGCAATCAAAACAA AATATTTTATTTTAGTTCAGTTTATTAAGTTATTATCA GTATCGTATTAAAAAATTAAAG ATCATTGAAAAATGTAAACCGATTATATTTTGTTTTTA AAGTAGATTATTATTAGAAAAT TATTAAGAGAATTCTGTGTTAAATTTATTGAAAGAGA AAATTTATTTTCCCTTATTAATT AAAGTCCTTTACTTTTTTTGAAAACTGTCAGTTTTTTG AAGAGTTATTTGTTTTGTTACC AATTGCTATCAGGTACCCGTAGAATTTTATTCAAGAT CTTTCCGTAACGGTTACCTTTCT GTCAAATTATC
uORF3 st-st fragment (pKP76)	CAAGGGCATCGGTCGACGGGGAATAAAGTGCATGA GCATACATCTTGAAAAAAAAAGATG AAAAATTTCCGACTTTAAATACGGAAGATAAATACTC CAACCTTTTTTTCCAATTCCGAA ATTTTAGTCTTCTTTAAAGAAGTTTCGGCTCGCTGTC TTACCTTTTAAAATCTTCTACTT CTTGACAGTACTTATCTTCTTATATAATAGATATACA AAACAAAACAAAACAAAAACTCA

bioRxiv preprint doi: https://doi.org/10.1101/2024.03.06.583729; this version posted March 7, 2024. The copyright holder for this preprint (which was not certified by peer review) is the author/funder. This article is a US Government work. It is not subject to copyright under 17 USC 105 and is also made available for use under a CC0 license.

	CAACACAGGTTACTCTCCCCCCTAAATTCAAATTTTT<br>TTTGCCCATCAGTTTCACTAGCG<br>AATTATACAACTCACCAGCCACACAGCTCACTCATC<br>TACTTCGCAATCAAAACAAAATAT<br>TTTATTTTAGTTCAGTTTATTAAGTTATTATCAGTATC<br>GTATTAAAAAATTAAAGATCAT<br>TGAAAAAAGCTTGCTAAACCGATTATATTTTGTTTTT<br>AAAGTAGATTATTATTAGAAAAT<br>TATTAAGAGAATTCTGTGTTAAATTTATTGAAAGAGA<br>AAATTTATTTTCCCTTATTAATT<br>AAAGTCCTTTACTTTTTTTGAAAACTGTCAGTTTTTTG<br>AAGAGTTATTTGTTTTGTTACC<br>AATTGCTATCATGTAGAATTTTATTCAAGATCTTTCC<br>GTAACGGTTACCTTTCTGTCAAA
uORF4 st-st fragment (pKP77)	CAAGGGCATCGGTCGACGGGGAATAAAGTGCATGA<br>GCATACATCTTGAAAAAAAAAGATG<br>AAAAATTTCCGACTTTAAATACGGAAGATAAATACTC<br>CAACCTTTTTTTCCAATTCCGAA<br>ATTTTAGTCTTCTTTAAAGAAGTTTCGGCTCGCTGTC<br>TTACCTTTTAAAATCTTCTACTT<br>CTTGACAGTACTTATCTTCTTATATAATAGATATACA<br>AAACAAAACAAAACAAAAACTCA<br>CAACACAGGTTACTCTCCCCCCTAAATTCAAATTTTT<br>TTTGCCCATCAGTTTCACTAGCG<br>AATTATACAACTCACCAGCCACACAGCTCACTCATC<br>TACTTCGCAATCAAAACAAAATAT<br>TTTATTTTAGTTCAGTTTATTAAGTTATTATCAGTATC<br>GTATTAAAAAATTAAAGATCAT<br>TGAAAAAAGCTTGCTAAACCGATTATATTTTGTTTTT<br>AAAGTAGATTATTATTAGAAAAT<br>TATTAAGAGAATTCTGTGTTAAATTTATTGAAAGAGA<br>AAATTTATTTTCCCTTATTAATT<br>AAAGTCCTTTACTTTTTTTGAAAACTGTCAGTTTTTTG<br>AAGAGTTATTTGTTTTGTTACC<br>AATTGCTATCAGGTACCCGTAGAATTTTATTCAAGAT<br>GTAACGGTTACCTTTCTGTCAAA
*Sal*I-*Bst*EII fragment of pSG61 with WT uORF1 underlined. For pSG61-pSG89, the third (TGC) codon is replaced with other codons.	GTCGACCCCGTCCTGTGGATCTTCGGGGAATAAAGTGCA<br>TGAGCATACATCTTGAAAAAAAAAGATGAAAAATTTCCGA<br>CTTTAAATACGGAAGATAAATACTCCAACCTTTTTTTCCAA<br>TTCCGAAATTTTAGTCTTCTTTAAAGAAGTTTCGGCTCGC<br>TGTCTTACCTTTTAAAATCTTCTACTTCTTGACAGTACTTA<br>TCTTCTTATATAATAGATATACAAAACAAAACAAAACAAAA<br>ACTCACAACACAGGTTACTCTCCCCCCTAAATTCAAATTT<br>TTTTTGCCCATCAGTTTCACTAGCGAATTATACAACTCAC

bioRxiv preprint doi: https://doi.org/10.1101/2024.03.06.583729; this version posted March 7, 2024. The copyright holder for this preprint (which was not certified by peer review) is the author/funder. This article is a US Government work. It is not subject to copyright under 17 USC 105 and is also made available for use under a CC0 license.

	CAGCCACACAGCTCACTCATCTACTTCGCAATCAAAACAA AATATTTTATTTTAGTTCAGTTTATTAAGTTATTATCAGTAT CGTATTAAAAAATTAAAGATCATTGAAAAATGGCTTGCTA AACCGATTATATTTTGTTTTTAAAGTAGATTATTATTAGAA AATTATTAAGAGAATTCTGTGTTAAATTTATTGAAAGAGAA AATTTATTTTCCCTTATTAATTAAAGTCCTTTACTTTTTTTG AAAACTGTCAGTTTTTTGAAGAGTTATTTGTTTTGTTACCA ATTGCTATCAGGTACCCGTAGAATTTTATTCAAGAGGTTT CCGTAACGGTTACCTTTCTGTCAAATTATCCAGGTTTACT CGCCAATAAAAATTTCCCTATACTATCATTAATTAAATCAT TATTATTACTAAAGTTTTGTTTACC

bioRxiv preprint doi: https://doi.org/10.1101/2024.03.06.583729; this version posted March 7, 2024. The copyright holder for this preprint (which was not certified by peer review) is the author/funder. This article is a US Government work. It is not subject to copyright under 17 USC 105 and is also made available for use under a CC0 license.

SUPPLEMENTARY REFERENCES

1. Young DJ*, et al.* Tma64/eIF2D, Tma20/MCT-1, and Tma22/DENR Recycle Post-termination 40S Subunits In Vivo. *Mol Cell* **71**, 761-774 e765 (2018).
2. Mueller PP, Harashima S, Hinnebusch AG. A segment of *GCN4* mRNA containing the upstream AUG codons confers translational control upon a heterologous yeast transcript. *Proc Natl Acad Sci USA* **84**, 2863-2867 (1987).
3. Gunisova S, Beznoskova P, Mohammad MP, Vlckova V, Valasek LS. In-depth analysis of cis-determinants that either promote or inhibit reinitiation on GCN4 mRNA after translation of its four short uORFs. *RNA* **22**, 542-558 (2016).
4. Longtine MS*, et al.* Additonal modules for versatile and economical PCR-based gene deletion and modification in *Saccharomyces cerevisiae*. *Yeast* **14**, 953-961 (1998).
5. Carter Z, Delneri D. New generation of loxP-mutated deletion cassettes for the genetic manipulation of yeast natural isolates. *Yeast* **27**, 765-775 (2010).
6. Gunisova S, Valasek LS. Fail-safe mechanism of GCN4 translational control-uORF2 promotes reinitiation by analogous mechanism to uORF1 and thus secures its key role in GCN4 expression. *Nucleic Acids Res* **42**, 5880-5893 (2014).
7. Grant CM, Hinnebusch AG. Effect of sequence context at stop codons on efficiency of reinitiation in *GCN4* translational control. *Mol Cell Biol* **14**, 606-618 (1994).
